# Treatment pathways and clinical outcomes in newly diagnosed multiple myeloma outside Europe and North America: The INTEGRATE study

**DOI:** 10.1007/s12185-025-03972-8

**Published:** 2025-04-15

**Authors:** Kihyun Kim, Estelle Verburgh, Tatiana Mitina, Wenming Chen, Su-Peng Yeh, Natalia Schutz, Fahad Alsharif, Wee Joo Chng, Zhongwen Huang, Meral Beksac

**Affiliations:** 1 Samsung Medical Center, Sungkyunkwan University School of Medicine, Seoul, Korea; 2 Groote Schuur Hospital, University of Cape Town, Cape Town, South Africa; 3 Moscow Regional Research Clinical Institute Named After M.F. Vladimirsky, Moscow, Russia; 4 Beijing Chaoyang Hospital, Capital Medical University, Beijing, China; 5 China Medical University Hospital, Taichung, Taiwan; 6 Hospital Italiano de Buenos Aires, Buenos Aires, Argentina; 7 King Faisal Specialist Hospital and Research Center, Riyadh, Saudi Arabia; 8 National University Cancer Institute Singapore (NCIS), Singapore, Singapore; 9 Takeda Pharmaceuticals International Co., Cambridge, USA; 10 Ankara University Department of Hematology, Cebeci Hospital, Balkiraz, Mamak Cd. No:12, 06620 Mamak, Ankara Turkey

**Keywords:** Hematological malignancy, Treatment outcomes, Developing countries, Stem cell transplantation, Real-world evidence

## Abstract

**Background:**

Real-world data on multiple myeloma (MM) outside Europe and North America are limited. The INTEGRATE study retrospectively assessed real-world treatment pathways and outcomes in MM from Argentina, China, South Korea, South Africa, Russia, Saudi Arabia, Taiwan, and Türkiye.

**Methods:**

Medical records (2010–2011) of patients (≥ 18 years) with newly diagnosed MM were analyzed. The primary endpoint was time to next treatment (TTNT). Secondary endpoints included treatment pathways and clinical outcomes stratified by stem cell transplantation (SCT).

**Results:**

Of 1511 patients analyzed (median age: 59.5 years), 32% had IgG kappa MM and 35.9% had International Staging System stage III disease. Bortezomib- and thalidomide-based chemotherapy regimens were the most common first- and second-line treatments; lenalidomide-based regimens were common in later lines. Median TTNT from initiation of first-line treatment was 39.5 months. Only 31.7% of patients received SCT at diagnosis, with improved outcomes versus those without SCT (median overall survival: 114.1 vs 85.9 months; 5-year relapse-free rates after first-line treatment: 58.2% vs 49.3%).

**Conclusion:**

Treatment strategies for MM outside Europe and North America align with guideline recommendations. More effective treatments and SCT at treatment initiation are needed. This study can guide future research in these regions utilizing newer treatment options.

**Supplementary Information:**

The online version contains supplementary material available at 10.1007/s12185-025-03972-8.

## Introduction

Multiple myeloma (MM), the second most common hematological malignancy, affects plasma cells [1, 2]. The discovery of new immunomodulatory drugs and proteasome inhibitors (e.g., carfilzomib, ixazomib, pomalidomide, etc.) in the last decade has significantly changed the treatment landscape in MM [1, 2]. Additionally, novel therapies like CAR-T cell therapies and monoclonal antibodies have further improved outcomes in MM [3]. However, such developments have also added complexity to the selection of an optimal treatment strategy at first and subsequent lines, as well as the best sequence of agents to use.

Despite the availability of novel agents, MM remains mostly incurable, with several challenges including disease heterogeneity, delayed diagnosis, poor outcomes, and patient resistance to multiple drug classes [3]. The unclear influence of disease symptoms and treatment-related toxicities on the use of effective novel therapies, especially in later lines of treatment, identifying the most efficient combination of novel agents in induction and maintenance regimens, and defining the best therapeutic strategies according to risk stratification and patient characteristics, are unmet challenges in MM management [3, 4]. In developing regions, these challenges are further complicated by limited access to novel treatments, often due to cost and resource limitations [5, 6].

Information on the current real-world management of MM is limited, especially in regions outside Europe and North America [7–12]. The available real-world studies from developing regions are limited by sample sizes, center participation, and data quality [13–16]. The INTEGRATE study was conducted in patients with MM to collect information on diagnosis, treatment pathways, the proportion of stem cell transplantation (SCT), and real-world outcomes in regions outside Europe and North America. Data from patients with newly diagnosed MM (NDMM) are presented here.

## Materials and methods

### Study design and population

The INTEGRATE study is an international, multicenter, observational, retrospective study of patients with MM. Forty-five sites from eight countries (Argentina, China, Republic of Korea, Republic of South Africa, Russia, Saudi Arabia, Taiwan, and Türkiye) were included. China, Republic of Korea, and Taiwan were grouped together as East Asia for the purpose of this analysis. Participating centers were selected based on physician interest, resource availability, start-up information, and accessibility to a defined minimum dataset (patient characteristics, diagnosis, treatment information, clinical outcomes, and adverse events [AEs]) for all patients. All centers were required to have specialist MM treatment departments, access to complete treatment journey of patients, appropriate personnel to collect data, electronic data capture system, adequate sample size, and adequate means to systematically identify eligible patients for study inclusion. The study was conducted in accordance with the Declaration of Helsinki and the International Conference on Harmonization Guideline for Good Clinical Practice. The relevant independent ethics committees/institutional review boards at each center approved the study and patients provided written informed consent (Supplementary methods).

All included patients were ≥ 18 years, alive or deceased at the time of retrospective data extraction, and diagnosed with MM between January 1, 2010, and December 31, 2011 (Supplementary material S1A). All patients were required to have completed at least one full line of treatment. Patients with smoldering myeloma, monoclonal gammopathy of unknown significance, participation in an interventional clinical trial during the study period, or without the minimum study dataset were excluded.

Pseudo-anonymized data were collected from March 21, 2018, to March 29, 2021, using a standardized web-based electronic case report form and assessed for random selection, pooled analysis, and reporting. Data were collected from the date of diagnosis until the death of the patient or the date when the patient was last known to be alive, whichever occurred first.

### Study endpoints

The primary endpoint of this study was time to next treatment (TTNT) in patients with MM. TTNT was defined as the time (in months) from the date of initiation of each line of treatment to the date of initiation of the next line of treatment or death in each patient.

Secondary endpoints included patients undergoing and not undergoing SCT, median duration of treatment, median overall survival (OS), best clinical response to each line of treatment according to the International Myeloma Working Group 2016 criteria, documented relapse or disease progression rates after start of first-line treatment, OS and relapse-free rates at 1, 2, and 5 years after start of first-line treatment and SCT, and AEs (assessed by severity and line of treatment).

### Statistical analysis

The results were presented for the overall sample and at individual country/regional level. Baseline characteristics, treatment pathways, clinical outcomes, and AEs were presented as summary statistics. Categorical endpoints were summarized with frequency distributions (number, %) using only non-missing data for a particular variable. Continuous variables were summarized using measures of central tendency (median) and spread (range or interquartile range [IQR]). The TTNT after each line of treatment was summarized using the Kaplan–Meier method. The association between TTNT or death with time and the potential covariates/factors were assessed using the Cox model, where hazard ratios, the respective 95% confidence intervals (CI), and P-values were presented. All analyses were conducted using Statistical Analysis System (SAS^®^) Software, Version 9.4 (SAS Institute Inc., Cary, NC, USA).

## Results

Of the 2087 enrolled patients, 1855 were eligible for the study and 213 were excluded due to ineligibility (Supplementary material S1B). A total of 1511 patients with NDMM (Table 1) involving 59 from Argentina, 565 from East Asia, 387 from Russia, 48 from Saudi Arabia, 104 from South Africa, and 348 from Türkiye (Supplementary material S1B) were analyzed. The remaining patients were diagnosed with relapsed/refractory MM (RRMM) during the study period and were analyzed separately, whose results are outside the scope of this manuscript.

**Table 1:** Demographic and baseline characteristics by SCT status in patients with NDMM

Characteristic^a^	Overall (N = 1511)	SCT (n = 479)	Non-SCT (n = 987)
Median age at MM diagnosis, years (range)	59.5 (24.4–97.3)	54.7 (24.4–73.8)	63.1 (26.8–97.3)
Median age at first-line treatment	59.6 (24.5–97.3)	54.8 (24.5–73.8)	63.1 (26.9–97.3)
Gender, n (%)			
Male	765 (50.6)	261 (54.5)	478 (48.4)
Ethnicity, n (%)			
White	758 (50.2)	207 (43.2)	518 (52.5)
Asian	572 (37.9)	169 (35.3)	396 (40.1)
Black or African American	51 (3.4)	15 (3.1)	34 (3.4)
Other^b^	67 (4.4)	37 (7.7)	28 (2.8)
Type of myeloma, n (%)			
IgG kappa	484 (32.0)	142 (29.6)	332 (33.6)
IgG lambda	239 (15.8)	77 (16.1)	159 (16.1)
IgG (light chain unknown)	55 (3.6)	11 (2.3)	43 (4.4)
IgA kappa	148 (9.8)	43 (9.0)	104 (10.5)
IgA lambda	97 (6.4)	37 (7.7)	58 (5.9)
IgA (light chain unknown)	13 (0.9)	1 (0.2)	12 (1.2)
IgD	10 (0.7)	4 (0.8)	6 (0.6)
IgM	14 (0.9)	2 (0.4)	12 (1.2)
IgE	0	0	0
Light chain alone (kappa or lambda)	235 (15.6)	82 (17.1)	151 (15.3)
ISS stage at diagnosis, n (%)			
Stage I	193 (18.4)	88 (22.9)	100 (15.9)
Stage II	293 (27.9)	100 (26.0)	189 (30.1)
Stage III	377 (35.9)	119 (30.9)	252 (40.1)
Unknown	185 (17.6)	78 (20.3)	84 (13.4)
Patients with plasmacytoma, n (%)	314 (20.8)	116 (24.2)	195 (19.8)
Patients meeting criteria for CRAB, n (%)	1508 (99.8)	476 (99.4)	987 (100.0)
Calcium elevated, n (%)	101 (6.7)	38 (8.0)	62 (6.3)
Renal failure, n (%)	246 (16.3)	78 (16.4)	163 (16.5)
Anemia, n (%)	867 (57.5)	235 (49.4)	609 (61.7)
Any bone lesions, n (%)	1231 (81.6)	388 (81.5)	811 (82.2)
0 sites	185 (15.0)	49 (12.7)	131 (16.1)
1–3 sites	533 (43.3)	182 (47.0)	336 (41.4)
> 3 sites	452 (36.7)	127 (32.8)	321 (39.5)

For 45 patients with NDMM, the status of SCT at MM diagnosis was reported as ‘unknown’. Therefore, the data for these 45 patients are not included in the SCT or non-SCT cohorts

*CRAB* calcium (elevated), renal failure, anemia, bone lesions, *IgA* immunoglobulin A, *IgD* immunoglobulin D, *IgE* immunoglobulin E, *IgG* immunoglobulin G, *IgM* immunoglobulin M, *ISS* International Staging System, *MM* multiple myeloma, *NDMM* newly diagnosed multiple myeloma, *SCT* stem cell transplantation

^a^Baseline characteristics were recorded at the time of MM diagnosis

^b^Others include American Indian or Alaska Native, Native Hawaiian or another Pacific Islander, and other ethnicities

### Patient baseline characteristics

Overall, the median age at MM diagnosis was 59.5 years, IgG kappa was the most common type of myeloma (32.0%), International Staging System (ISS) Stage III disease was the most common stage (35.9%), and most patients had bone lesions (81.5%) (Table 1). Most patient baseline characteristics were similar across the different countries (Supplementary material S2). Of note, the proportion of females was higher than males in Argentina (54.2%) and Russia (59.7%), most patients in Argentina had ISS Stage I disease (45.3%), and a higher proportion of patients in East Asia had only light chain myeloma (24.2%).

### Treatment pathways

#### First-line SCT

**Fig. 1:**
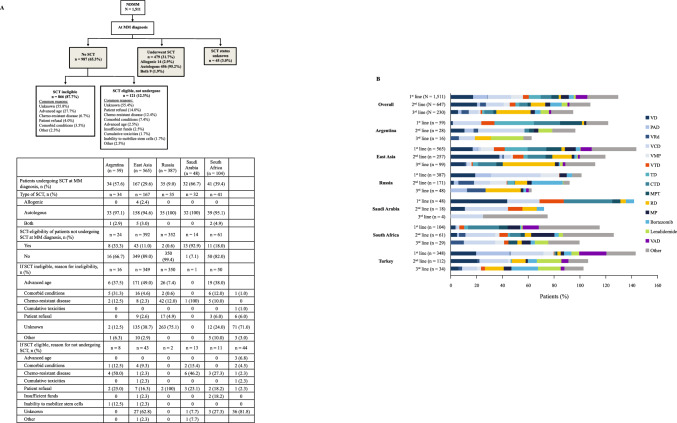
Treatment patterns in patients with NDMM. **A** SCT pattern during MM diagnosis, overall (flowchart) and by region (table). **B** Most common (frequency ≥ 10%) chemotherapy regimens in the first three lines of treatment, overall and by region. *CTD* cyclophosphamide, thalidomide, dexamethasone, *MM* multiple myeloma, *MP* melphalan, prednisone, *MPT* melphalan, thalidomide, prednisone, *NDMM* newly diagnosed multiple myeloma, *PAD* bortezomib, doxorubicin, dexamethasone, *RD* lenalidomide, dexamethasone, *SCT* stem cell transplantation, *TD* thalidomide, dexamethasone, *VAD* vincristine, doxorubicin, dexamethasone, *VCD* bortezomib, cyclophosphamide, dexamethasone, *VD* bortezomib, dexamethasone, *VMP* bortezomib, melphalan, prednisone, *VRd* bortezomib, lenalidomide, dexamethasone, *VTD* bortezomib, thalidomide, dexamethasone

Only 479 (31.7%) patients of the 1511 patients with NDMM underwent SCT, while 987 (65.3%) did not have SCT and 45 (3.0%) reported an undisclosed status regarding SCT (Fig. 1A). The proportion of patients with NDMM undergoing first-line SCT was highest in Saudi Arabia (32/48, 66.7%) followed by Argentina (34/59, 57.6%), and was lowest in Russia (35/387, 9.0%). Of the 987 patients who did not have first-line SCT, most were SCT-ineligible (87.7%). The reason for SCT ineligibility was not known in 55.8% of the SCT-ineligible patients with NDMM, while it was advanced age in most of the remaining SCT-ineligible patients. Additionally, 12.3% of SCT-eligible patients did not undergo first-line SCT, although the reason for this was unknown in 55.4% of patients. Patient refusal and chemo-resistance were the next most common reasons for patients to not receive SCT.

#### Treatment distribution

All patients with NDMM received first-line treatment. However, the proportion of patients receiving subsequent lines of treatment decreased with each subsequent line of therapy (Fig. 1B). Bortezomib- and thalidomide-based chemotherapy regimens were the most common first- and second-line treatments, respectively (Fig. 1B). Bortezomib, cyclophosphamide, plus dexamethasone (VCD) and bortezomib plus dexamethasone (VD) were the most common first- (18.3%) and second-line regimens (20.4%), respectively. Lenalidomide-based regimens (lenalidomide and dexamethasone [RD]) were the most common chemotherapy regimens in later lines of treatment. This observation was consistent across all participating regions except Argentina, where thalidomide-based regimens were more common during the earlier lines of treatment. Treatment completion and disease progression were reported as the two main reasons for treatment change after first-, second-, and third-line treatment. Additionally, toxicity was reported as a common reason (8.0%, n = 21) for treatment change during first-line treatment with VD in patients with NDMM. The overall median durations of treatment across the first three lines of treatment in patients with NDMM were generally similar (first-line: 6.0 [IQR: 3.7–10.4], second-line: 6.4 [IQR: 3.2–11.9], and third-line: 6.6 [IQR: 3.3–9.6] months) and this was generally consistent across the regions (Supplementary material S3).

### TTNT and treatment-free interval

**Fig. 2:**
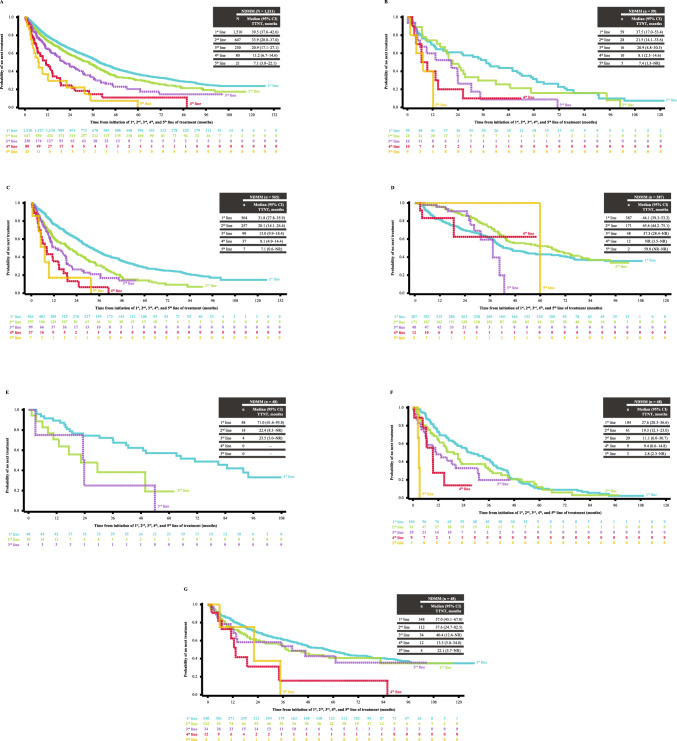
TTNT for different lines of treatment in patients with NDMM. **A** Overall, **B** Argentina, **C** East Asia, **D** Russia, **E** Saudi Arabia, **F** South Africa and **G** Türkiye. Patients who did not start the next line of treatment and were alive at that time were censored at the last known date to be alive at the time of data extraction. *CI* confidence interval, *NDMM* newly diagnosed multiple myeloma, *NR* not reached, *TTNT* time to next treatment

The overall median (95% CI) TTNT from initiation of first-line treatment in patients with NDMM was 39.5 (37.6–42.6) months (Fig. 2A) and the treatment-free interval (TFI) after first-line treatment was 33.5 months (Supplementary material S4). Median TTNT and TFI decreased with subsequent lines of treatment. The TFI between the first-line and second-line treatments were longer in patients undergoing SCT compared with non-SCT patients. Median TTNT from initiation of first-line treatment for NDMM varied across the regions and was lowest in South Africa for all lines of treatment (Fig. 2B–G). In a risk factor analysis, significantly shorter TTNT was observed in the following subgroups of patients with NDMM: Asian ethnicity, no SCT performed at first-line, and ISS Stage II/III (all P ≤ 0.022).

### Clinical outcomes

**Fig. 3:**
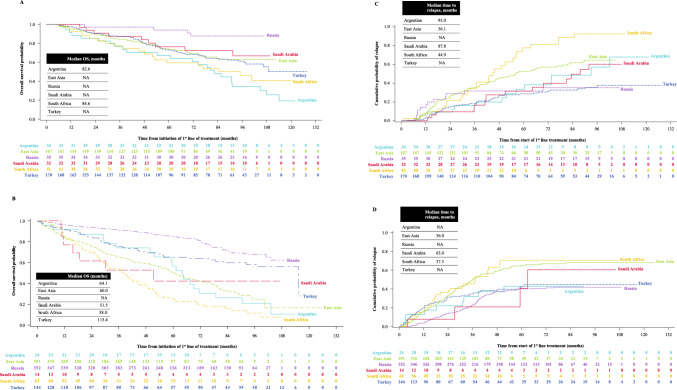
Overall survival and relapse probability in patients with NDMM by SCT status. **A** Overall survival in patients undergoing SCT. **B** Overall survival in non-SCT patients. **C** Probability of relapse in patients undergoing SCT. **D** Probability of relapse in non-SCT patients. *NA* not available, *NDMM* newly diagnosed multiple myeloma, *OS* overall survival, *SCT* stem cell transplantation

Most patients with NDMM achieved complete, very good partial, or partial responses after the first two lines of treatment (Table 2). The clinical responses tended to decline with subsequent lines of treatment. All clinical outcomes were better in patients undergoing SCT than in those without SCT. The 5-year OS and relapse-free rates after first-line treatment in patients undergoing SCT and those without SCT were 73.5% vs 64.6%, and 58.2% vs 49.3%, respectively. The median OS and median time to cumulative probability of relapse after first-line treatment were longer in patients undergoing SCT than in those without SCT (Fig. 3). The median OS in patients undergoing SCT was 114.1 months (IQR 55.7–NR) and in non-SCT patients was 85.9 months (IQR 38.7–NR). The clinical outcomes in the different regions were consistent with those of the overall results.

**Table 2:** Clinical outcomes by SCT status in patients with NDMM, overall and by region

	Overall (N = 1511)		Argentina (n = 59)		East Asia (n = 565)		Russia (n = 387)		Saudi Arabia (n = 48)		South Africa (n = 104)		Türkiye (n = 348)	
	SCT (n = 479)	Non-SCT (n = 987)	SCT (n = 34)	Non-SCT (n = 24)	SCT (n = 167)	Non-SCT (n = 392)	SCT (n = 35)	Non-SCT (n = 352)	SCT (n = 32)	Non-SCT (n = 14)	SCT (n = 41)	Non-SCT (n = 61)	SCT (n = 170)	Non-SCT (n = 144)
Best clinical response to first-line treatment, n (%)														
sCR	15 (3.1)	10 (1.0)	1 (2.9)	0	7 (4.2)	5 (1.3)	1 (2.9)	4 (1.1)	5 (15.6)	1 (7.1)	0	0	1 (0.6)	0
CR	146 (30.5)	195 (19.8)	4 (11.8)	3 (12.5)	48 (28.7)	66 (16.8)	18 (51.4)	78 (22.2)	23 (71.9)	2 (14.3)	2 (4.9)	10 (16.4)	51 (30.0)	36 (25.0)
VGPR	109 (22.8)	193 (19.6)	18 (52.9)	7 (29.2)	44 (26.3)	78 (19.9)	3 (8.6)	92 (26.1)	2 (6.3)	2 (14.3)	14 (34.1)	8 (13.1)	28 (16.5)	6 (4.2)
PR	138 (28.8)	245 (24.8)	8 (23.5)	8 (33.3)	55 (32.9)	113 (28.8)	7 (20.0)	77 (21.9)	2 (6.3)	2 (14.3)	22 (53.7)	13 (21.3)	44 (25.9)	32 (22.2)
MR	4 (0.8)	27 (2.7)	0	0	2 (1.2)	16 (4.1)	0	4 (1.1)	0	0	0	1 (1.6)	2 (1.2)	6 (4.2)
SD	22 (4.6)	136 (13.8)	2 (5.9)	3 (12.5)	8 (4.8)	55 (14.0)	1 (2.9)	62 (17.6)	0	1 (7.1)	2 (4.9)	3 (4.9)	9 (5.3)	12 (8.3)
PD	3 (0.6)	67 (6.8)	0	2 (8.3)	0	24 (6.1)	0	17 (4.8)	0	6 (42.9)	1 (2.4)	16 (26.2)	2 (1.2)	2 (1.4)
Best clinical response to second-line treatment, n (%)														
sCR	5 (2.5)	0	2 (14.3)	0	2 (2.2)	0	0	0	0	0	0	0	1 (2.1)	0
CR	52 (25.9)	87 (20.2)	2 (14.3)	1 (7.1)	23 (25.6)	14 (8.5)	4 (36.4)	53 (33.1)	5 (38.5)	2 (50.0)	5 (20.0)	2 (5.7)	13 (27.1)	15 (28.8)
VGPR	40 (19.9)	103 (24.0)	7 (50.0)	3 (21.4)	17 (18.9)	31 (18.8)	5 (45.5)	66 (41.3)	0	1 (25.0)	5 (20.0)	0	6 (12.5)	2 (3.8)
PR	42 (20.9)	78 (18.1)	0	2 (14.3)	24 (26.7)	35 (21.2)	1 (9.1)	24 (15.0)	4 (30.8)	0	9 (36.0)	13 (37.1)	4 (8.3)	4 (7.7)
MR	9 (4.5)	12 (2.8)	0	1 (7.1)	7 (7.8)	5 (3.0)	0	3 (1.9)	1 (7.7)	0	0	1 (2.9)	1 (2.1)	2 (3.8)
SD	12 (6.0)	36 (8.4)	1 (7.1)	3 (21.4)	6 (6.7)	24 (14.5)	0	6 (3.8)	0	0	5 (20.0)	3 (8.6)	0	0
PD	12 (6.0)	41 (9.5)	1 (7.1)	1 (7.1)	6 (6.7)	21 (12.7)	1 (9.1)	3 (1.9)	2 (15.4)	1 (25.0)	0	15 (42.9)	2 (4.2)	0
Best clinical response to third-line treatment, n (%)														
sCR	3 (3.8)	0	1 (9.1)	0	0	0	0	0	0	0	2 (11.1)	0	0	0
CR	13 (16.3)	47 (32.4)	1 (9.1)	1 (20.0)	5 (13.2)	4 (6.7)	1 (50.0)	36 (78.3)	0	0	4 (22.2)	0	2 (20.0)	6 (28.6)
VGPR	11 (13.8)	15 (10.3)	4 (36.4)	0	5 (13.2)	7 (11.7)	0	6 (13.0)	0	0	2 (11.1)	1 (9.1)	0	1 (4.8)
PR	20 (25.0)	22 (15.2)	1 (9.1)	2 (40.0)	13 (34.2)	17 (28.3)	0	1 ( 2.2)	1 (100)	0	5 (27.8)	2 (18.2)	0	0
MR	5 (6.3)	2 (1.4)	0	0	5 (13.2)	2 (3.3)	0	0	0	0	0	0	0	0
SD	6 (7.5)	16 (11.0)	1 (9.1)	1 (20.0)	3 (7.9)	8 (13.3)	0	2 (4.3)	0	0	2 (11.1)	5 (45.5)	0	0
PD	9 (11.3)	18 (12.4)	2 (18.2)	1 (20.0)	5 (13.2)	10 (16.7)	1 (50.0)	1 (2.2)	0	2 (100)	1 (5.6)	3 (27.3)	0	1 (4.8)
Patients with any documented clinical response from first-line treatment, n (%)	423 (88.3)	727 (73.7)	34 (100)	19 (79.2)	159 (95.2)	293 (74.7)	30 (85.7)	288 (81.8)	32 (100)	8 (57.1)	39 (95.1)	35 (57.4)	129 (75.9)	84 (58.3)
Median time difference between first response and first relapse/disease progression (IQR), months	24.7 (10.2–46.3)	10.8 (1.3–26.9)	47.7 (9.9–65.8)	1.0 (0.3–20.9)	22.3 (10.5–42.3)	5.7 (1.0–19.0)	7.9 (3.6–15.1)	23.6 (9.1–33.0)	39.0 (28.7–76.9)	26.8 (-8.5–59.2)	30.1 (16.6–48.5)	10.2 (1.0–25.3)	19.6 (9.1–41.1)	2.8 (1.3–18.5)
Any documented relapse or disease progression after first-line treatment, n (%)	207 (43.2)	434 (44.0)	14 (41.2)	10 (41.7)	89 (53.3)	184 (46.9)	12 (34.3)	138 (39.2)	15 (46.9)	9 (64.3)	28 (68.3)	42 (68.9)	49 (28.8)	51 (35.4)
Relapse-free rate post first-line treatment, % (95% CI)														
1-year	94.2 (91.7–96.0)	88.4 (86.1–90.3)	93.9 (77.9–98.4)	87.1 (65.2–95.7)	92.6 (87.3–95.7)	84.3 (79.8–87.8)	85.7 (69.0–93.8)	96.2 (93.6–97.8)	100 (100–100)	92.3 (56.6–98.9)	92.6 (78.7–97.5)	83.3 (71.1–90.6)	97.0 (92.9–98.7)	80.1 (72.2–86.0)
2-year	80.5 (76.5–83.8)	75.5 (72.4–78.3)	90.7 (73.8–96.9)	78.0 (54.8–90.2)	73.8 (66.2–80.0)	67.3 (61.6–72.5)	71.2 (53.1–83.4)	86.2 (82.0–89.5)	90.5 (73.4–96.8)	92.3 (56.6–98.9)	78.6 (61.5–88.7)	63.7 (49.4–75.0)	85.4 (78.8–90.0)	68.8 (59.6–76.4)
5-year	58.2 (53.2–62.9)	49.3 (45.4–53.1)	66.4 (44.6–81.3)	57.2 (33.4–75.3)	47.2 (38.8–55.1)	35.6 (29.1–42.0)	68.2 (50.0–81.0)	60.0 (54.0–65.4)	68.4 (47.8–82.2)	59.3 (15.7–86.3)	22.3 (9.30–38.8)	29.4 (15.5–44.8)	71.4 (63.1–78.2)	57.1 (46.5–66.3)
Relapse-free rate post first SCT, % (95% CI)														
1-year	85.2 (81.6–88.2)	–	90.3 (72.8–96.8)	-	79.6 (72.4–85.1)	-	77.1 (59.5–87.9)	-	93.6 (76.9–98.4)	-	88.9 (73.0–95.7)	-	89.1 (83.1–93.1)	-
2-year	74.3 (69.9–78.2)	–	83.1 (63.9–92.6)	-	66.2 (58.1–73.1)	-	68.2 (50.0–81.0)	-	86.9 (68.8–94.9)	-	68.2 (49.9–81.0)	-	81.1 (73.8–86.6)	-
5-year	54.9 (49.8–59.8)	–	60.4 (38.2–76.7)	-	45.4 (37.0–53.4)	-	65.0 (46.6–78.4)	-	60.2 (39.4–75.9)	-	21.7 (8.94–38.0)	-	68.3 (59.5–75.6)	-
Median OS (95% CI)	114.1 (106.2–NR)	85.9 (79.2–97.1)	82.6 (54.3–104.8)	64.1 (49.8–71.5)	NR	60.0 (54.7–68.1)	NR	NR	NR (91.4–NR)	51.4 (11.7–NR)	84.6 (52.0–NR)	38.0 (27.6–48.5)	NR (97.7– NR)	115.4 (82.1–NR)
OS rate after start of first-line treatment, % (95% CI)														
1-year	96.2 (94.0–97.6)	90.1 (88.1–91.9)	91.2 (75.1–97.1)	91.5 (70.0–97.8)	95.1 (90.5–97.5)	84.2 (80.0–87.6)	100 (100–100)	96.6 (94.1–98.0)	100 (100–100)	76.9 (44.2–91.9)	92.7 (79.0–97.6)	85.2 (73.6–92.0)	97.6 (93.8–99.1)	92.8 (87.1–96.1)
2-year	89.7 (86.6–92.1)	83.5 (81.0–85.8)	85.3 (68.2–93.6)	87.1 (65.2–95.7)	89.4 (83.5–93.3)	75.9 (71.0–80.1)	97.1 (80.9–99.6)	93.7 (90.6–95.8)	100 (100–100)	61.5 (30.8–81.8)	85.3 (70.2–93.1)	70.0 (56.6–79.9)	90.3 (84.6–93.9)	84.5 (76.9–89.7)
5-year	73.5 (69.1–77.4)	64.6 (61.2–67.9)	67.1 (48.4–80.2)	56.6 (34.4–73.8)	73.7 (65.9–80.0)	50.5 (44.2–56.4)	93.9 (77.8–98.4)	85.2 (80.8–88.6)	91.7 (53.9–98.8)	42.2 (14.9–67.7)	59.4 (41.5–73.4)	26.2 (15.7–38.0)	72.9 (65.0–79.3)	65.0 (55.2–73.1)

There were 45 patients with NDMM (Argentina: 1, East Asia: 6, Saudi Arabia: 2, South Africa: 2, Türkiye: 34), in whom the status of SCT at MM diagnosis was reported as ‘unknown’ and these are not included in the above table

*CI* confidence interval, *CR* complete response, *IQR* interquartile range, *MR* minimal response, *NDMM* newly diagnosed multiple myeloma, *NR* not reached, *OS* overall survival, *PD* progressive disease, *PR* partial response, *sCR* stringent complete response, *SCT* stem cell transplantation, *SD* stable disease, *VGPR* very good partial response

A comparative summary of the treatment regimen choices and clinical outcomes in the current study and other real-world studies from developed and developing regions is shown in Table 3.

**Table 3:** Comparison of the INTEGRATE study with other real-world studies in MM

Study	Study type	Study period	Study region	Follow-up duration	Patient type	Sample size	Age	Proportion of SCT	Treatment choice	Clinical outcomes
Developed regions: USA, Europe, Japan										
He et al. [12]	Retrospective review of US-SEER and Optum databases	2007–2018	US	NA	NDMM with no transplant	17,731	Mean: 71 years	0	Bortezomib- and lenalidomide-based	OS range: 56.3–112.6 months TTNT range: 7–24.3 months
Cejalvo et al. [18]	Retrospective analysis	NA	Spain	NA	Transplant-ineligible NDMM	675	Mean: 75.6 years	SCT-ineligible	First-line: non-VMP bortezomib-based	Median OS: 33.5 months (95% CI 29.1–37.2)
Remes et al. [20]	Retrospective, observational study of Finnish Hematology Registry	2009–2013	Finland	Median: 25 months	NDMM	321	Median: 66.0 years	NA	Conventional (n = 46) vs ASCT-novel (n = 114) vs non-SCT-novel (n = 161)	Median OS: Conventional: 25.6 months Non-SCT-novel: 46.2 months ASCT-novel: NR Median TTNT: Conventional: 7.8 months Non-SCT-novel: 12.6 months SCT-novel: 33.9 months
Hajek et al. [32]	Noninterventional, observational, retrospective analysis of prospectively collected patient medical record	2007–2014	Czech	NA	NDMM and RRMM	2446	NA	NA	Bortezomib-, thalidomide-, and lenalidomide-based	Median OS: 50.3 months OS, PFS, and depth of response decreased with subsequent lines of treatment
Mohty et al. [10]	Prospective, non-interventional study	2010–2012	Europe (several countries), Türkiye, Israel, South Africa	NA	NDMM and RRMM	2358	Median: 63.0 years	n = 775	Bortezomib-, thalidomide-, and lenalidomide-based	First-line bortezomib or thalidomide/lenalidomide: SCT: ORR: > 85%, VGPR: > 50% Non-SCT: ORR: 64–85%, VGPR: 24–53% First-line other regimen: SCT: ORR: 71%, VGPR: 29% Non-SCT: ORR: 51%, VGPR: 10%
Yong et al. [11]	Observational cross-sectional and retrospective patient chart review	2014	Belgium, France, Germany, Italy, Spain, Switzerland, and UK	12 months in the past year	MM	4997	> 65 years: 64%	Not stated	Bortezomib- and thalidomide-based regimens common in first-line and lenalidomide-based regimens in third-line Median duration of 1L treatment – 6 months	Median treatment-free interval: 10 months VGPR: 74% for 1L vs 11% for 5L Clinical outcomes better with SCT vs no SCT (P < 0.001 for treatment-free interval and complete response)
Akizuki et al. [17]	Retrospective study	2010–2018	Japan	32.8 months	NDMM	284	Median: 71 years	16.5%	80% received novel agents (51.8% received bortezomib-based regimens as first-line)	NA
Developing regions										
Hungria et al. [19]	Analysis of 2 observational studies datasets	1998–2007	Latin America and Asia	NA	MM	3664	NA	26%	NA	Median OS: 56 months in Latin America and 47 months in Asia (HR: 0.83, P < 0.001)
Hungria et al. [21, 35]	Retrospective medical chart review	2008–2015	Latin America	Median 2.2 years	NDMM	1518	Median: 61.0 years	33.9%	Thalidomide- and bortezomib-based regimens	Median PFS following first-line treatment SCT: 31.1 months Non-SCT: 15 months Median PFS following second-line treatment: SCT: 9.5 months Non-SCT: 10.9 months PR or above: 91.7% with SCT vs 64.9% without SCT
Hungria et al. [28]	Retrospective-prospective study	2007–2009	Argentina, Brazil, Chile, Mexico, and Peru	62 months	MM	852	Mean (SCT-eligible:): 54.7 years Mean (SCT-ineligible): 67.4 years	ASCT: 26.9%	Thalidomide-based regimens most common in SCT-ineligible patients	Median OS: SCT-ineligible: 43 months SCT: 73.6 months
Nasr et al. [24]	Retrospective study	2002–2019	Lebanon	NA	NDMM	134	Mean: 61.9 years	24.6%	Bortezomib-based regimens were most common induction agents	Mean PFS similar across different bortezomib-based regimens Mean PFS with SCT vs no SCT (75 vs 52 months, P = 0.016)
Qian et al. [15]	Retrospective study	2007–2015	China	NA	NDMM	122	Median: 70.5 years	NA	Both novel and conventional agents used	Median OS: 33 months 5-year OS rate: 30.4% OS with and without bortezomib-based regimens: 37 vs 28 months
Weil et al. [36]	Retrospective study	2009–2015	Israel	12 months	NDMM	552	Mean: 65.6 years	38.4%	Both novel and conventional agents used	Median OS: 5.2 months Median OS with bortezomib-based regimens: 6.5 months

*1L* first-line, *5L* fifth-line, *ASCT* autologous stem cell transplantation, *CR* complete remission, *HR* hazard ratio, *MM* multiple myeloma, *NA* not available, *NDMM* newly diagnosed multiple myeloma, *NR* not reached, *ORR* objective response rate, *OS* overall survival, *PFS* progression-free survival, *PR* partial response, *RRMM* relapsed/refractory multiple myeloma, *SCT* stem cell transplantation, *TTNT* time to next treatment, *US-SEER* United States-Surveillance, Epidemiology, and End Results Program, *VGPR* very good partial remission, *VMP* bortezomib, melphalan, prednisone

### Adverse events

For a median follow-up duration of 57 months, a total of 1623 AEs were reported in 579 (38.3%) patients with NDMM. Of these, 599 were treatment-related AEs reported in 274 (18.1%) patients, and 478 were serious adverse events reported in 267 (17.7%) patients.

## Discussion

INTEGRATE is one of the largest real-world studies to characterize the management of MM in regions outside Europe and North America. It showed that novel agents like proteasome inhibitors and immunomodulatory drugs in combination with first-line SCT during earlier lines of treatment led to better clinical outcomes across regions like Argentina, East Asia, Russia, Saudi Arabia, South Africa, and Türkiye. Considering the limited real-world evidence regarding MM in these regions, the present study contributes relevant real-world data to the literature. Additionally, these findings may help serve as reference for future studies on MM in these regions.

In general, patient characteristics in patients with NDMM were similar to those reported in other real-world studies [5–14, 16–21]. Although consistent with previous real-world reports [8, 17, 19, 22–25], the SCT rates at MM diagnosis in this study was low (31.7%), despite evidence suggesting early SCT following induction therapy in eligible patients with MM is the standard of care to achieve deep response [26]. Most patients who did not receive SCT were ineligible, although the reason for this was not recorded in over half of the ineligible patients. Among the SCT-eligible patients, patient refusal and comorbid conditions were cited as causal factors for not receiving SCT treatment. The logistical challenges associated with low numbers of transplant centers, waiting lists, as well as added costs in resource-limited countries [5, 6, 27], could also have contributed to the lower proportion of SCT observed in these regions.

Patients with NDMM most commonly received bortezomib-based regimens for the first three lines and lenalidomide-based regimens for later lines of treatment across all regions except Argentina. This is consistent with other real-world studies from both developed and developing countries [9–12, 15, 17–20, 24]. In Argentina, thalidomide-based regimens were common in the first two lines of treatment, consistent with previous observations in Latin America [27–29]. The observed use of the same treatments in different lines is in contrast with current treatment patterns [30], where clinicians opt to change drugs for different lines of therapy. Consistent with observations from other real-world studies [9–12, 15, 17–20, 24], choice of chemotherapy regimens varied considerably in clinical practice across the different regions (Fig. 1B and Supplementary material S3). Although triplet regimens were more common than doublet regimens during first-line treatment, their use declined with subsequent lines of treatment. This may be due to poor tolerance, drug resistance, decreased performance status of patients with disease progression, limited accessibility to triplet regimens, or higher costs.

The treatment regimens were generally used for similar durations to those reported in other real-world studies [9, 11, 18, 20, 31, 32]. Completion of planned treatment was a common reason for treatment change, which contrasts with current practices [30], where treatment continues until disease progression. This could be because maintenance treatments were not common at the time of the study, or treatment durations in developing regions were only for a fixed duration due to costs and/or accessibility. The proportion of patients receiving subsequent lines of treatment consistently decreased with each line of treatment, which was consistent with other real-world studies [8, 11, 25]. This may be due to frailty, advanced age, loss to follow-up, and complications or death associated with disease severity. In resource-limited countries, this may also be influenced by increased costs and complexity of treatment regimens and patients’ ability to pay for treatment [5, 6, 33]. The decreasing proportion of patients receiving subsequent lines of treatment suggests the need for upfront use of effective and optimal treatment strategies, rather than reserving them for later lines.

The median TTNT in patients with NDMM, especially for the first three lines of treatment was relatively longer (range 20.9–39.5 months) than found in other real-world studies of MM with novel agents (range 12.6–38.4 months) [10, 12, 20, 34]. The OS and clinical response rates in our study were higher than those reported in other real-world studies [6, 8–11, 19, 20, 28, 32]. A US study by Mohty et al. showed a median TTNT ranging from 19.3 to 38.4 months with lenalidomide- and bortezomib-based regimens [9], and a retrospective study in China showed better median OS with bortezomib-based regimens than with conventional regimens (37 vs 28 months, P = 0.029) [15]. Median OS and clinical response rates were significantly better in patients with first-line SCT than in the non-SCT cohort. This is consistent with other real-world studies that have shown the positive impact of SCT on clinical outcomes in patients with NDMM [8, 20, 28]. In a multicenter registry study in Finland, the median TTNT for first-line treatment (33.9 months) and median OS (not reached) with novel agents combined with SCT were better than without SCT (12.6 and 46.2 months, respectively) [20]. The Latin American HOLA study showed that patients with SCT had longer median OS (79.3 vs 52.8 months, P < 0.0001), longer median PFS following first-line treatment (31.1 vs 15.0 months, P < 0.0001), and higher rates of partial response or above (91.7% vs 64.9%, P < 0.0001) than patients without SCT [35]. A European study and a Latin American study showed that patients with SCT had a higher complete response at first-line (47% vs 25% of patients; P < 0.001) [11] and better OS (73.6 vs 43.0 months) [28] than patients without SCT, respectively. Further, any documented relapse or disease progression after first-line treatment for both patients undergoing SCT and those without SCT were less than 50%, implying that some patients may have altered their treatment regimens even without clinically evident progression meeting International Myeloma Working Group (IMWG) criteria, as observed by the TTNT of 39.5 months within the first-line population. Together, these findings may suggest that the clinical outcomes may be better with the use of novel agents and SCT during the earlier lines of treatment in patients with NDMM.

This study has several limitations due to its retrospective nature. The patients were predominantly from specialized treatment centers, thus may not represent the entire patient population. Treatment pathways and clinical outcomes were based on investigators’ assessments, and not on any conventionally defined criteria. The results may have been influenced by selection bias stemming from the retention of patients with minimal data, the inclusion of only patients with prior treatment, and the potential exclusion of older patients, potentially due to systematic bias in referrals to participating centers or case inclusions. The generalization of these data should be made with caution, due to the unknown impact of country- and institution-specific differences in availability, access, and choice of different lines of treatments, patient selection bias, and relatively younger and healthier patients than other real-world studies. Low patient numbers in later treatment lines due to deficiencies in the follow-up data limit the generalizability of the findings. However, this can be mitigated through careful interpretation, particularly when considering the sample sizes in each treatment line, such as focusing on the first three lines of therapy. The treatment choices presented here do not consider the physician’s intention underlying that treatment choice. The TTNT may not accurately reflect treatment effectiveness since the reasons for starting a new therapy are not always related to disease progression and may vary between different centers. Additionally, some patients who may have progressed to relapse or refractory MM during the study period may have influenced the overall results. The AE data presented here may only include those recorded in the medical records, and may not include AEs not formally recorded. Other limitations include not assessing progression-free survival and not assessing TTNT by SCT status. Therefore, these results should be interpreted with caution. Finally, the study findings are from 2010 to 2011, which may not be applicable today due to the changed diagnostic and treatment landscape in MM. However, these findings contribute important real-world data in MM from regions under-represented in literature.

In conclusion, our study provides a wealth of information to better understand the management of NDMM in regions outside Europe and North America. These retrospective real-world data highlight the diversity of treatments used to manage MM in clinical practice. Clinical outcomes were better in patients who underwent SCT than those without SCT, emphasizing a positive impact of SCT on long-term clinical outcomes. The variety of treatment regimens used in this study highlights that the treatment choices for different lines of therapy in MM should be individualized and strategically sequenced based on patient and tolerability profiles. The treatment strategy should consider the most effective and novel treatments at initiation of therapy for longer durations and using SCT earlier in treatment to achieve better outcomes. The findings of our study provide a rich source of real-world evidence for future research in MM, which can inform how best to meet the future needs of patients with MM, especially in regions outside Europe and North America.

## Supplementary Information

Below is the link to the electronic supplementary material.

Supplementary file1 (DOCX 288 KB)

## Data Availability

The datasets, including the redacted study protocol, redacted statistical analysis plan, and individual participants’ data supporting the results reported in this article, will be made available within 3 months from initial request to researchers who provide a methodologically sound proposal. The data will be provided after de-identification, in compliance with applicable privacy laws, data protection, and requirements for consent and anonymization.
