# Correction: Treatment pathways and clinical outcomes in newly diagnosed multiple myeloma outside Europe and North America: The INTEGRATE study

**DOI:** 10.1007/s12185-025-04012-1

**Published:** 2025-05-20

**Authors:** Kihyun Kim, Estelle Verburgh, Tatiana Mitina, Wenming Chen, Su-Peng Yeh, Natalia Schutz, Fahad Alsharif, Wee Joo Chng, Zhongwen Huang, Meral Beksac

**Affiliations:** 1 Samsung Medical Center, Sungkyunkwan University School of Medicine, Seoul, Korea; 2 Groote Schuur Hospital, University of Cape Town, Cape Town, South Africa; 3 Moscow Regional Research Clinical Institute Named After M.F. Vladimirsky, Moscow, Russia; 4 Beijing Chaoyang Hospital, Capital Medical University, Beijing, China; 5 China Medical University Hospital, Taichung, Taiwan; 6 Hospital Italiano de Buenos Aires, Buenos Aires, Argentina; 7 King Faisal Specialist Hospital and Research Center, Riyadh, Saudi Arabia; 8 National University Cancer Institute Singapore (NCIS), Singapore, Singapore; 9 Takeda Pharmaceuticals International Co., Cambridge, USA; 10 Ankara University Department of Hematology, Cebeci Hospital, Balkiraz, Mamak Cd. No:12, 06620 Mamak, Ankara Turkey

**Correction: International Journal of Hematology** 10.1007/s12185-025-03972-8

The article ‘Treatment pathways and clinical outcomes in newly diagnosed multiple myeloma outside Europe and North America: The INTEGRATE study’, written by Kihyun Kim, Estelle Verburgh, Tatiana Mitina, Wenming Chen, Su‑Peng Yeh, Natalia Schutz, Fahad Alsharif, Wee Joo Chng, Zhongwen Huang, Meral Beksac, was originally published under exclusive license to The Author(s), under exclusive licence to Japanese Society of Hematology 2025. As a result of the subsequent decision to publish the article under the open access model, the article’s copyright notice was changed on 05 May 2025 to © The Author(s) 2025 and the article is now distributed under a Creative Commons Attribution 4.0 International License, which permits use, sharing, adaptation, distribution and reproduction in any medium or format, as long as you give appropriate credit to the original author(s) and the source, provide a link to the Creative Commons licence, and indicate if changes were made. The images or other third party material in this article are included in the article's Creative Commons licence, unless indicated otherwise in a credit line to the material. If material is not included in the article's Creative Commons licence and your intended use is not permitted by statutory regulation or exceeds the permitted use, you will need to obtain permission directly from the copyright holder. To view a copy of this licence, visit http://creativecommons.org/licenses/by/4.0/.

