# Supplementary material for: Treatment pathways and clinical outcomes in newly diagnosed multiple myeloma outside Europe and North America: The INTEGRATE study
Source: Int J Hematol. 2025 Apr 15;122(2):231–46. doi: 10.1007/s12185-025-03972-8 (PMC12304004; doi:10.1007/s12185-025-03972-8)

**Treatment pathways and clinical outcomes in newly diagnosed multiple myeloma outside Europe and North America: The INTEGRATE study**

**Supplementary material**

**Supplementary methods**

Recruited patients were approached to provide written informed consent for their medical records to be reviewed for study data collection. A waiver was sought from ethics committees wherever possible to seek consent to access the medical records of deceased, alive or lost to follow-up patients for the purpose of this study.

The responsible site principal investigator and delegated site team provided the patient (and, if applicable, parent or legal guardian) with oral and written information about the study in a form that the patient (parent or legal guardian) could understand, and obtained the patient’s (and, if applicable, the patient’s legal guardian’s) written consent before identifiable patient information (hereinafter referred to as personal data) was collected. Before consenting, the patient (and, if applicable, legal guardian) was given ample time to consider and to ask questions. Since the study is observational, the consent only concerned the collection of data from medical charts and no further interventional procedures or treatments are required.

The patient had to agree that sponsor personnel, their representatives, or Independent Ethics

Committees (IECs)/Institutional Review Boards (IRBs) or Competent Authority (CA) personnel (national or other) may require direct access to the patient’s data/personal records that were collected, processed, and stored in an anonymous form. The patient had to agree that his/her data were processed and stored in an anonymous form for evaluation of this study and any later overviews, that data may also be transferred in anonymous form to third parties, e.g., other companies or authorities, and that data may be located in other countries with potentially different regulations.

The patient or legal guardian, if applicable, had the right to withdraw his/her consent at any time without prejudice. In the informed consent form (ICF) it is stated that, if consent was withdrawn, any data collected before withdrawal of consent will be kept. The original, signed ICFs were kept at the site.

**Supplementary material S1 (A) Study design and (B) Patient disposition.**

^a^The most common reasons for ineligibility were non-availability of minimum dataset from hospital medical records, no diagnosis of NDMM between January 1, 2010, and December 31, 2011, and patients not completing at least one full line of treatment.

*Observational period: The period from the date of NDMM diagnosis, between January 1, 2010 and December 31, 2011, until the date of death or the date when the patient was last known to be alive, whichever occurred first.

**Date of NDMM diagnosis: The date of NDMM entered in the case report form (CRF).

***Data abstraction time period: The time period when potentially eligible patient’s charts were retrieved from medical records and transcribed into the CRF.

**A**

^^

^^

**B**

**Supplementary material S2. Demographic and baseline characteristics by SCT status and by region in patients with NDMM.**

| **Characteristic^a^** | **Argentina** | | | **East Asia** | | | **Russia** | | | **Saudi Arabia** | | | **South Africa** | | | **Türkiye** | | |
| --- | --- | --- | --- | --- | --- | --- | --- | --- | --- | --- | --- | --- | --- | --- | --- | --- | --- | --- |
|  | **Overall**  **(n = 59)** | **SCT**  **(n = 34)** | **Non-SCT**  **(n = 24)** | **Overall**  **(n = 565)** | **SCT**  **(n = 167)** | **Non-SCT**  **(n = 392)** | **Overall**  **(n = 387)** | **SCT**  **(n = 35)** | **Non-SCT**  **(n = 352)** | **Overall**  **(n = 48)** | **SCT**  **(n = 32)** | **Non-SCT**  **(n = 14)** | **Overall**  **(n = 104)** | **SCT**  **(n = 41)** | **Non-SCT**  **(n = 61)** | **Overall**  **(n = 348)** | **SCT**  **(n = 170)** | **Non-SCT**  **(n = 144)** |
| Median age at MM diagnosis, years (range) | 58.9  (41.5–83.8) | 55.9  (41.5–66.8) | 68.1  (46.2–83.8) | 61.5  (30.0–97.3) | 55.6  (34.6–65.6) | 65.7  (30.0–97.3) | 60.5  (26.8–88.5) | 48.6  (41.9–62.4) | 61.6  (26.8–88.5) | 52.0 (29.4–81.5) | 52.0 (36.6–69.7) | 51.5 (29.4–81.5) | 56.7 (36.2–76.0) | 54.4 (38.5–66.9) | 60.0 (36.2-76.0) | 57.6 (24.4–85.0) | 55.2 (24.4–73.8) | 64.8 (35.6–85.0) |
| Male | 27 (45.8) | 13 (38.2) | 13 (54.2) | 309 (54.7) | 95 (56.9) | 210 (53.6) | 156 (40.3) | 18 (51.4) | 138 (39.2) | 30 (62.5) | 21 (65.6) | 8 (57.1) | 57 (54.8) | 22 (53.7) | 33 (54.1) | 186 (53.4) | 92 (54.1) | 76 (52.8) |
| Ethnicity, n (%)^b^ |  |  |  |  |  |  |  |  |  |  |  |  |  |  |  |  |  |  |
| Asian | 0 | 0 | 0 | 564 (99.8) | 167 (100) | 391 (99.7) | 0 | 0 | 0 | 0 | 0 | 0 | 7 (6.7) | 2 (4.9) | 5 (8.2) | 1 (0.3) | 0 | 0 |
| White | 39 (66.1) | 23 (67.6) | 15 (62.5) | 0 | 0 | 0 | 387 (100) | 35 (100) | 352 (100) | 0 | 0 | 0 | 29 (27.9) | 18 (43.9) | 11 (18.0) | 303 (87.1) | 131 (77.1) | 140 (97.2) |
| Black or African American | 0 | 0 | 0 | 0 | 0 | 0 | 0 | 0 | 0 | 0 | 0 | 0 | 51 (49.0) | 15 (36.6) | 34 (55.7) | 0 | 0 | 0 |
| Others^c^ | 2 (3.4) | 0 | 2 (8.3) | 1 (0.2) | 0 | 1 (0.3) | 0 | 0 | 0 | 48 (100) | 32 (100) | 14 (100) | 16 (15.4) | 5 (12.2) | 11 (18.0) | 0 | 0 | 0 |
| Type of myeloma, n (%) |  |  |  |  |  |  |  |  |  |  |  |  |  |  |  |  |  |  |
| IgG kappa | 25 (42.4) | 14 (41.2) | 10 (41.7) | 141 (25.0) | 42 (25.1) | 97 (24.7) | 167 (43.2) | 13 (37.1) | 154 (43.8) | 13 (27.1) | 7 (21.9) | 6 (42.9) | 41 (39.4) | 25 (61.0) | 16 (26.2) | 97 (27.9) | 41 (24.1) | 49 (34.0) |
| IgG lambda | 14 (23.7) | 11 (32.4) | 3 (12.5) | 107 (18.9) | 31 (18.6) | 76 (19.4) | 54 (14.0) | 4 (11.4) | 50 (14.2) | 6 (12.5) | 5 (15.6) | 0 | 15 (14.4) | 5 (12.2) | 10 (16.4) | 43 (12.4) | 21 (12.4) | 20 (13.9) |
| IgG (light chain unknown) | 2 (3.4) | 2 (5.9) | 0 | 12 (2.1) | 2 (1.2) | 10 (2.6) | 34 (8.8) | 5 (14.3) | 29 (8.2) | 2 (4.2) | 1 (3.1) | 0 | 1 (1.0) | 1 (2.4) | 0 | 4 (1.1) | 0 | 4 (2.8) |
| IgA kappa | 3 (5.1) | 1 (2.9) | 2 (8.3) | 57 (10.1) | 14 (8.4) | 43 (11.0) | 43 (11.1) | 4 (11.4) | 39 (11.1) | 6 (12.5) | 5 (15.6) | 1 (7.1) | 12 (11.5) | 3 (7.3) | 9 (14.8) | 27 (7.8) | 16 (9.4) | 10 (6.9) |
| IgA lambda | 2 (3.4) | 0 | 2 (8.3) | 57 (10.1) | 20 (12.0) | 35 (8.9) | 16 (4.1) | 1 (2.9) | 15 (4.3) | 3 (6.3) | 3 (9.4) | 0 | 4 (3.8) | 3 (7.3) | 1 (1.6) | 15 (4.3) | 10 (5.9) | 5 (3.5) |
| IgA (light chain unknown) | 0 | 0 | 0 | 6 (1.1) | 0 | 6 (1.5) | 7 (1.8) | 1 (2.9) | 6 (1.7) | 0 | 0 | 0 | 0 | 0 | 0 | 0 | 0 | 0 |
| IgD | 0 | 0 | 0 | 4 (0.7) | 2 (1.2) | 2 (0.5) | 5 (1.3) | 2 (5.7) | 3 (0.9) | 0 | 0 | 0 | 0 | 0 | 0 | 1 (0.3) | 0 | 1 (0.7) |
| IgM | 1 (1.7) | 0 | 1 (4.2) | 7 (1.2) | 2 (1.2) | 5 (1.3) | 4 (1.0) | 0 | 4 (1.1) | 0 | 0 | 0 | 2 (1.9) | 0 | 2 (3.3) | 0 | 0 | 0 |
| IgE | 0 | 0 | 0 | 0 | 0 | 0 | 0 | 0 | 0 | 0 | 0 | 0 | 0 | 0 | 0 | 0 | 0 | 0 |
| Light chain alone (kappa or lambda) | 11 (18.6) | 5 (14.7) | 6 (25.0) | 137 (24.2) | 48 (28.7) | 88 (22.4) | 24 (6.2) | 1 (2.9) | 23 (6.5) | 14 (29.2) | 9 (28.1) | 5 (35.7) | 12 (11.5) | 4 (9.8) | 8 (13.1) | 37 (10.6) | 15 (8.8) | 21 (14.6) |
| ISS stage at diagnosis, n (%) |  |  |  |  |  |  |  |  |  |  |  |  |  |  |  |  |  |  |
| Stage I | 24 (45.3) | 18 (54.5) | 6 (31.6) | 103 (19.9) | 42 (27.1) | 60 (16.8) | 14 (16.5) | 1 (33.3) | 13 (15.9) | 2 (4.2) | 1 (3.1) | 1 (7.1) | 4 (10.3) | 1 (20.0) | 3 (9.4) | 46 (14.9) | 25 (15.9) | 17 (13.8) |
| Stage II | 20 (37.7) | 10 (30.3) | 10 (52.6) | 169 (32.6) | 44 (28.4) | 123 (34.4) | 24 (28.2) | 2 (66.7) | 22 (26.8) | 3 (6.3) | 2 (6.3) | 1 (7.1) | 6 (15.4) | 1 (20.0) | 5 (15.6) | 71 (23.1) | 41 (26.1) | 28 (22.8) |
| Stage III | 8 (15.1) | 5 (15.2) | 3 (15.8) | 216 (41.7) | 67 (43.2) | 149 (41.6) | 47 (55.3) | 0 | 47 (57.3) | 11 (22.9) | 10 (31.3) | 1 (7.1) | 25 (64.1) | 3 (60.0) | 20 (62.5) | 70 (22.7) | 34 (21.7) | 32 (26.0) |
| Unknown | 1 (1.9) | 0 | 0 | 27 (5.2) | 2 (1.3) | 23 (6.4) | 0 | 0 | 0 | 32 (66.7) | 19 (59.4) | 11 (78.6) | 4 (10.3) | 0 | 4 (12.5) | 121 (39.3) | 57 (36.3) | 46 (37.4) |
| Patients with plasmacytoma, n (%) | 6 (10.2) | 5 (14.7) | 1 (4.2) | 155 (27.4) | 40 (24.0) | 113 (28.8) | 51 (13.2) | 8 (22.9) | 43 (12.2) | 13 (27.1) | 9 (28.1) | 4 (28.6) | 31 (29.8) | 13 (31.7) | 18 (29.5) | 58 (16.7) | 41 (24.1) | 16 (11.1) |
| Patients with bone lesions, n (%) | 34 (57.6) | 20 (58.8) | 14 (58.3) | 451 (79.8) | 136 (81.4) | 309 (78.8) | 363 (93.8) | 35 (100) | 328 (93.2) | 45 (93.8) | 31 (96.9) | 13 (92.9) | 94 (90.4) | 38 (92.7) | 54 (88.5) | 244 (70.1) | 127 (74.7) | 94 (65.3) |
| Patients with number of bone lesion sites, n (%) |  |  |  |  |  |  |  |  |  |  |  |  |  |  |  |  |  |  |
|  |  |  |  |  |  |  |  |  |  |  |  |  |  |  |  |  |  |  |
|  |  |  |  |  |  |  |  |  |  |  |  |  |  |  |  |  |  |  |
|  |  |  |  |  |  |  |  |  |  |  |  |  |  |  |  |  |  |  |
| Patients meeting criteria for CRAB, n (%) | 59 (100) | 34 (100) | 24 (100) | 563 (99.6) | 165 (98.8) | 392 (100) | 387 (100) | 35 (100) | 352 (100) | 48 (100) | 32 (100) | 14 (100) | 103 (99.0) | 40 (97.6) | 61 (100) | 348 (100) | 170 (100) | 144 (!00) |
| Calcium elevated, n (%) | 2 (3.4) | 1 (2.9) | 1 (4.2) | 34 (6.0) | 14 (8.5) | 19 (4.8) | 24 (6.2) | 5 (14.3) | 19 (5.4) | 13 (27.1) | 6 (18.8) | 7 (50.0) | 8 (7.8) | 2 (5.0) | 6 (9.8) | 20 (5.7) | 10 (5.9) | 10 (6.9) |
| Renal failure, n (%) | 17 (28.8) | 10 (29.4) | 7 (29.2) | 112 (19.9) | 33 (20.0) | 78 (19.9) | 51 (13.2) | 6 (17.1) | 45 (12.8) | 9 (18.8) | 7 (21.9) | 2 (14.3) | 14 (13.6) | 5 (12.5) | 9 (14.8) | 43 (12.4) | 17 (10.0) | 22 (15.3) |
| Anemia, n (%) | 38 (64.4) | 20 (58.8) | 17 (70.8) | 372 (66.1) | 96 (58.2) | 274 (69.9) | 217 (56.1) | 18 (51.4) | 199 (56.5) | 18 (37.5) | 10 (31.3) | 6 (42.9) | 45 (43.7) | 18 (45.0) | 27 (44.3) | 177 (50.9) | 73 (42.9) | 86 (59.7) |
| Any bone lesions, n (%) | 34 (57.6) | 20 (58.8) | 14 (58.3) | 454 (80.6) | 136 (82.4) | 312 (79.6) | 363 (93.8) | 35 (100) | 328 (93.2) | 45 (93.8) | 31 (96.9) | 13 (92.9) | 94 (91.3) | 38 (95.0) | 54 (88.5) | 241 (69.3) | 128 (75.3) | 90 (62.5) |
| 0 sites | 1 (2.9) | 0 | 1 (7.1) | 77 (17.1) | 18 (13.2) | 58 (18.8) | 40 (11.0) | 3 (8.6) | 37 (11.3) | 4 (8.9) | 3 (9.7) | 0 | 10 (10.6) | 3 (7.9) | 7 (13.0) | 53 (21.7) | 22 (17.3) | 28 (29.8) |
| 1–3 sites | 19 (55.9) | 9 (45.0) | 10 (71.4) | 233 (51.7) | 75 (55.1) | 154 (49.8) | 113 (31.1) | 10 (28.6) | 103 (31.4) | 16 (35.6) | 10 (32.3) | 6 (46.2) | 37 (39.4) | 11 (28.9) | 24 (44.4) | 115 (47.1) | 67 (52.8) | 39 (41.5) |
| >3 sites | 7 (20.6) | 6 (30.0) | 1 (7.1) | 136 (30.2) | 42 (30.9) | 93 (30.1) | 207 (57.0) | 19 (54.3) | 188 (57.3) | 25 (55.6) | 18 (58.1) | 7 (53.8) | 45 (47.9) | 23 (60.5) | 22 (40.7) | 32 (13.1) | 19 (15.0) | 10 (10.6) |

CRAB = calcium (elevated), renal failure, anemia, bone lesions; IgA = immunoglobulin A; IgD = immunoglobulin D; IgE = immunoglobulin E; IgG = immunoglobulin G; IgM = immunoglobulin M; ISS = International Staging System; MM = multiple myeloma; NDMM = newly diagnosed multiple myeloma; SCT = stem cell transplantation.

^a^The baseline characteristics were recorded at the time of MM diagnosis.

^b^Ethnicity was not reported in 18 patients in Argentina, 1 patient in South Africa, and 44 patients in Türkiye.

^c^Others include American Indian or Alaska Native, Native Hawaiian or another Pacific Islander, and Other.

**Supplementary material S3. Treatment duration with the most common regimens (frequency ≥10% in any of the first three lines of treatment in any region) in patients with NDMM, overall and by region.**

|  | Overall  (N = 1,511) | | | Argentina  (n = 59) | | | East Asia  (n = 565) | | | Russia  (n = 387) | | | Saudi Arabia  (n = 48) | | | South Africa  (n = 104) | | | Türkiye  (n = 348) | | |
| --- | --- | --- | --- | --- | --- | --- | --- | --- | --- | --- | --- | --- | --- | --- | --- | --- | --- | --- | --- | --- | --- |
| Median duration of treatment, (IQR) months | 1^st^ line  (N = 1,511) | 2^nd^ line  (N = 647) | 3^rd^ line  (N = 230) | 1^st^ line  (n = 59) | 2^nd^ line  (n = 28) | 3^rd^ line  (n = 16) | 1^st^ line  (n = 565) | 2^nd^ line  (n = 257) | 3^rd^ line  (n = 99) | 1^st^ line  (n = 387) | 2^nd^ line  (n = 171) | 3^rd^ line  (n = 48) | 1^st^ line  (n = 48) | 2^nd^ line  (n = 18) | 3^rd^ line  (n = 4) | 1^st^ line  (n = 104) | 2^nd^ line  (n = 61) | 3^rd^ line  (n = 29) | 1^st^ line  (n = 348) | 2^nd^ line  (n = 112) | 3^rd^ line  (n = 34) |
| **Overall** | 6.0  (3.7–10.4) | 6.4  (3.2–11.9) | 6.6  (3.3–9.6) | 6.7  (3.2–18.0) | 4.1  (2.5–12.6) | 7.8  (4.4–21.6) | 6.4  (3.3–14.7) | 6.1  (2.3–11.9) | 6.7  (2.8–10.7) | 6.0  (4.6–7.1) | 6.7  (5.8–12.0) | 6.7  (6.5–7.6) | 5.8  (3.9–7.8) | 7.6  (3.7–13.0) | 7.0  (4.0–9.0) | 7.4  (5.4–11.7) | 6.5  (3.6–12.2) | 5.4  (3.0–12.1) | 5.1  (3.0–9.7) | 5.0  (2.7–9.3) | 3.4  (2.0–7.0) |
| **Bortezomib-based** | | | | | | | | | | | | | | | | | | | | | |
| VD | 4.3  (2.4–6.4) | 3.6  (1.9–7.6) | 3.3  (1.0–5.9) | 4.5  (4.5–4.5) | 3.4  (2.5–10.2) | 0 | 2.5  (1.6–4.1) | 3.3  (1.6–7.6) | 3.4  (2.0–6.7) | 6.0  (4.7–6.4) | 5.2  (3.2–7.2) | 0 | 4.6  (4.0–6.2) | 23.2 (13.0–33.5) | 0 | 8.4  (2.2–15.1) | 9.3  (4.6–13.8) | 0 | 4.0  (2.7–7.0) | 4.0  (3.0–6.7) | 0.6  (0.3–1.0) |
| PAD | 3.8  (2.0–5.1) | 4.0  (1.7–4.9) | 6.5  (5.6–6.9) | 0 | 4.0  (4.0–4.0) | 0 | 3.9  (1.7–5.0) | 3.1  (0.8–5.4) | 0 | 3.9  (3.0–5.2) | 4.6  (4.0–4.9) | 6.5  (5.6–6.9) | 0 | 0 | 0 | 4.0  (4.0–4.0) | 2.9  (2.9–2.9) | 0 | 3.4  (1.8–5.1) | 0 | 0 |
| VRd | 3.3  (1.1–7.2) | 6.7  (4.1–8.0) | 6.7  (3.7–7.5) | 0 | 13.5  (13.5–13.5) | 7.5  (7.5–7.5) | 1.1  (1.1–1.1) | 4.2  (0.9–28.2) | 0.6  (0.6–0.6) | 0 | 6.7  (5.8–8.0) | 6.8  (6.5–7.7) | 0 | 0 | 0 | 0 | 7.2  (2.8–8.7) | 3.0  (2.0–7.9) | 5.2  (3.3–7.2) | 3.9  (1.8–5.9) | 3.7  (3.7–3.7) |
| VCD | 5.2  (3.7–6.3) | 4.6  (2.9–6.5) | 4.7  (3.0–5.4) | 3.7  (2.6–7.2) | 4.1  (2.6–4.4) | 5.5  (3.5–7.6) | 2.9  (0.7–4.4) | 5.0  (1.3–10.5) | 1.4  (0.4–4.4) | 6.0  (4.9–6.9) | 5.8  (4.8–8.6) | 0 | 3.9  (3.0–6.1) | 5.5  (3.5–8.9) | 8.8  (8.8–8.8) | 4.2  (3.3–5.2) | 4.7  (2.6–5.2) | 4.9  (4.7–5.5) | 4.0  (3.0–5.4) | 3.9  (2.7–5.0) | 3.1  (2.9–4.3) |
| VMP | 7.2  (4.0–11.6) | 7.8  (6.7–13.4) | 2.3  (1.1–3.1) | 0 | 0 | 0 | 6.1  (2.3–11.4) | 3.5  (0.3–9.1) | 0.4  (0.4–0.4) | 8.3  (6.0–15.1) | 8.2  (7.1–14.1) | 0 | 0 | 0 | 0 | 0 | 2.1  (1.4–2.8) | 2.3  (1.7–3.0) | 6.5  (3.9–10.0) | 7.3  (6.8–7.9) | 3.3  (3.3–3.3) |
| VTD | 3.7  (2.3–6.5) | 4.2  (1.4–8.6) | 5.7  (1.3–7.6) | 5.4  (3.2–8.0) | 0 | 0 | 3.6  (2.2–5.2) | 3.3  (1.3–9.1) | 0 | 8.0  (8.0–8.0) | 6.9  (6.9–6.9) | 0 | 3.3  (1.9–5.1) | 6.5  (1.3–11.8) | 0 | 5.2  (3.4–15.5) | 4.3  (2.6–6.1) | 5.7  (5.7–5.7) | 4.8  (3.0–6.5) | 0 | 1.3  (1.3–1.3) |
| **Thalidomide-based** | | | | | | | | | | | | | | | | | | | | | |
| TD | 3.3  (2.2–7.0) | 12.1  (10.8–17.0) | 3.5  (1.5–4.1) | 6.2  (3.2–8.6) | 0 | 0 | 3.2  (2.0–6.9) | 10.0  (6.4–13.3) | 0 | 0 | 12.2  (12.1–24.0) | 0 | 3.4  (2.6–6.2) | 0 | 0 | 6.4  (3.9–7.0) | 49.1  (49.1–49.1) | 0 | 1.6  (0.9–2.4) | 14.1 (14.1–14.1) | 0 |
| CTD | 3.3  (2.8–5.1) | 5.6  (2.9–9.6) | 6.3  (2.5–9.0) | 3.3  (3.2–3.8) | 25.8  (25.8–25.8) | 0 | 3.0  (2.7–4.2) | 5.3  (2.5–8.6) | 6.3  (2.8–10.1) | 0 | 12.0  (12.0–12.0) | 0 | 4.5  (1.5–5.1) | 0 | 0 | 5.0  (3.8–7.0) | 6.0  (5.0–15.1) | 3.4  (0.0–6.9) | 3.2  (3.2–3.2) | 0 | 0 |
| MPT | 6.2  (2.9–11.2) | 4.2  (0.9–8.1) | 1.8  (1.1–2.2) | 10.1  (3.0–18.0) | 6.2  (6.2–6.2) | 0 | 5.5  (2.1–10.1) | 3.0  (0.7–8.0) | 1.8  (1.1–2.2) | 0 | 0 | 0 | 0 | 0 | 0 | 5.7  (4.8–11.6) | 6.4  (4.6–8.2) | 0 | 11.8  (6.1–15.0) | 0 | 0 |
| **Lenalidomide-based** | | | | | | | | | | | | | | | | | | | | | |
| RD | 8.4  (3.8–26.0) | 9.2  (4.7–16.3) | 6.7  (3.9–10.7) | 18.9 (18.9–18.9) | 0 | 6.8  (0.7–12.8) | 10.1  (3.0–32.4) | 8.0  (3.2–14.9) | 5.2  (3.1–11.0) | 0 | 12.2  (8.9–18.7) | 6.7  (6.6–7.1) | 2.2  (0.7–3.8) | 31.9  (1.7–62.2) | 0 | 0 | 5.0  (1.1–5.7) | 15.3 (15.3–15.3) | 4.9  (4.0–26.0) | 12.7  (7.7–26.4) | 9.0  (7.0–12.6) |
| **Melphalan-based** | | | | | | | | | | | | | | | | | | | | | |
| MP | 5.8  (2.6–11.8) | 10.4  (4.1–13.8) | 2.0  (0.6–8.5) | 0 | 3.8  (3.8–3.8) | 0 | 5.3  (1.2–13.9) | 13.8  (1.8–16.5) | 0.1  (0.1–1.1) | 4.9  (3.3–7.2) | 12.6  (12.0–18.7) | 10.3  (10.3–10.3) | 0 | 0 | 0 | 7.6  (7.1–11.8) | 8.6  (4.1–12.7) | 2.0  (1.6–2.3) | 5.9  (2.6–9.4) | 5.7  (1.0–10.4) | 8.5  (7.5–9.6) |
| **Others** | | | | | | | | | | | | | | | | | | | | | |
| Bortezomib | 4.6  (2.8–8.0) | 6.2  (3.8–6.6) | 2.4  (1.9–7.6) | 0 | 0 | 0 | 4.2  (1.9–9.5) | 0.9  (0.4–2.8) | 8.8  (8.8–8.8) | 5.8  (5.2–10.2) | 6.4  (6.2–6.7) | 9.4  (9.4–9.4) | 1.9  (1.7–2.2) | 0.5  (0.5–0.5) | 0 | 11.3  (11.3–11.3) | 10.0  (8.9–11.1) | 7.6  (7.6–7.6) | 3.5  (2.5–5.5) | 2.5  (1.0–3.3) | 2.3  (1.8–2.4) |
| Lenalidomide | 22.6  (9.7–30.1) | 10.0  (4.0–28.1) | 12.7  (4.0–16.2) | 0 | 30.3  (21.6–36.8) | 15.4  (11.0–43.2) | 5.9  (2.6–9.2) | 34.0  (0.2–67.9) | 23.9  (14.1–33.7) | 0 | 6.6  (6.6–6.6) | 23.9  (14.1–33.7) | 0 | 0 | 0 | 67.3  (67.3–67.3) | 33.2  (28.1–38.4) | 4.0  (3.7–7.8) | 23.2 (10.9–29.8) | 7.3  (3.8–17.9) | 8.4  (2.0–15.0) |
| VAD | 3.0  (2.0–5.2) | 2.7  (2.1–3.3) | 3.1  (1.3–4.9) | 0 | 0 | 7.8  (4.4–21.6) | 2.6  (1.2–3.1) | 3.3  (2.7–4.1) | 0 | 0 | 0 | 6.7  (6.5–7.6) | 0 | 0 | 0 | 5.9  (4.1–6.4) | 0 | 0 | 3.5  (2.0–5.5) | 2.2  (1.8–3.0) | 2.4  (2.4–2.4) |

Note: In patients with NDMM, the only regimen with ≥10% frequency in fourth-line treatment was lenalidomide and dexamethasone combination (13.8%) for a median duration of 6.5 months. None of the fifth-line treatments had a frequency ≥5%.

CTD = cyclophosphamide, thalidomide, dexamethasone; IQR = interquartile range; MP = melphalan, prednisone; MPT = melphalan, thalidomide, prednisone;
NDMM = newly diagnosed multiple myeloma; PAD = bortezomib, doxorubicin, dexamethasone; RD = lenalidomide, dexamethasone; TD = thalidomide, dexamethasone; VAD = vincristine, doxorubicin, dexamethasone; VCD = bortezomib, cyclophosphamide, dexamethasone; VD = bortezomib, dexamethasone; VMP = bortezomib, melphalan, prednisone; VRd = bortezomib, lenalidomide, dexamethasone; VTD = bortezomib, thalidomide, dexamethasone.

**Supplementary material S4.** **Overall treatment duration and treatment-free interval after first three lines of treatment in patients with NDMM**

**A) All patients**

**
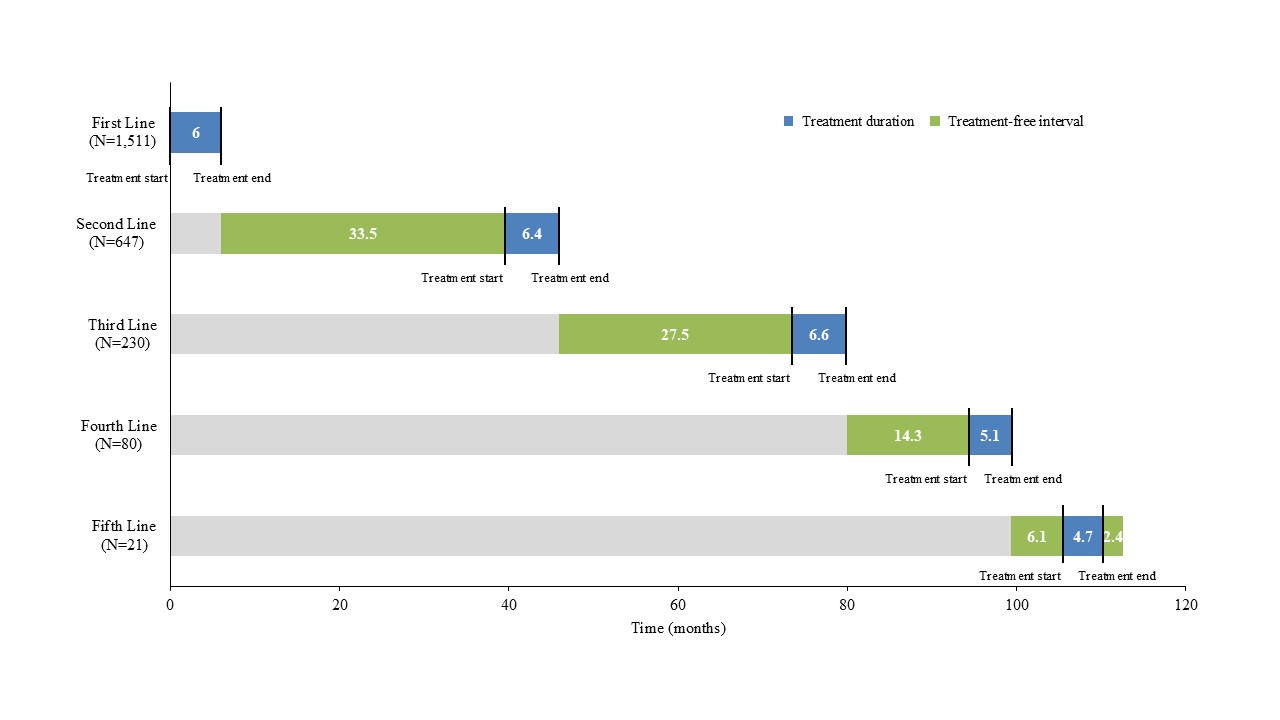
**

**B) Patients undergoing SCT**


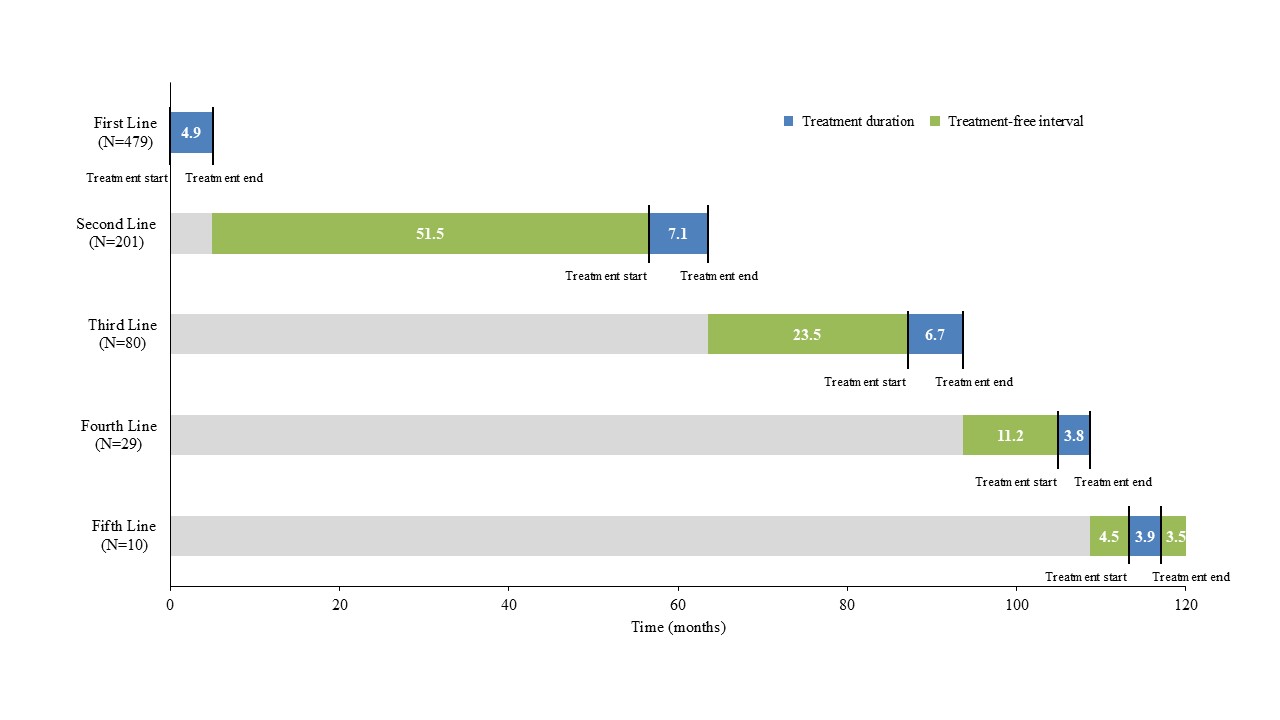


**C) Non-SCT patients**


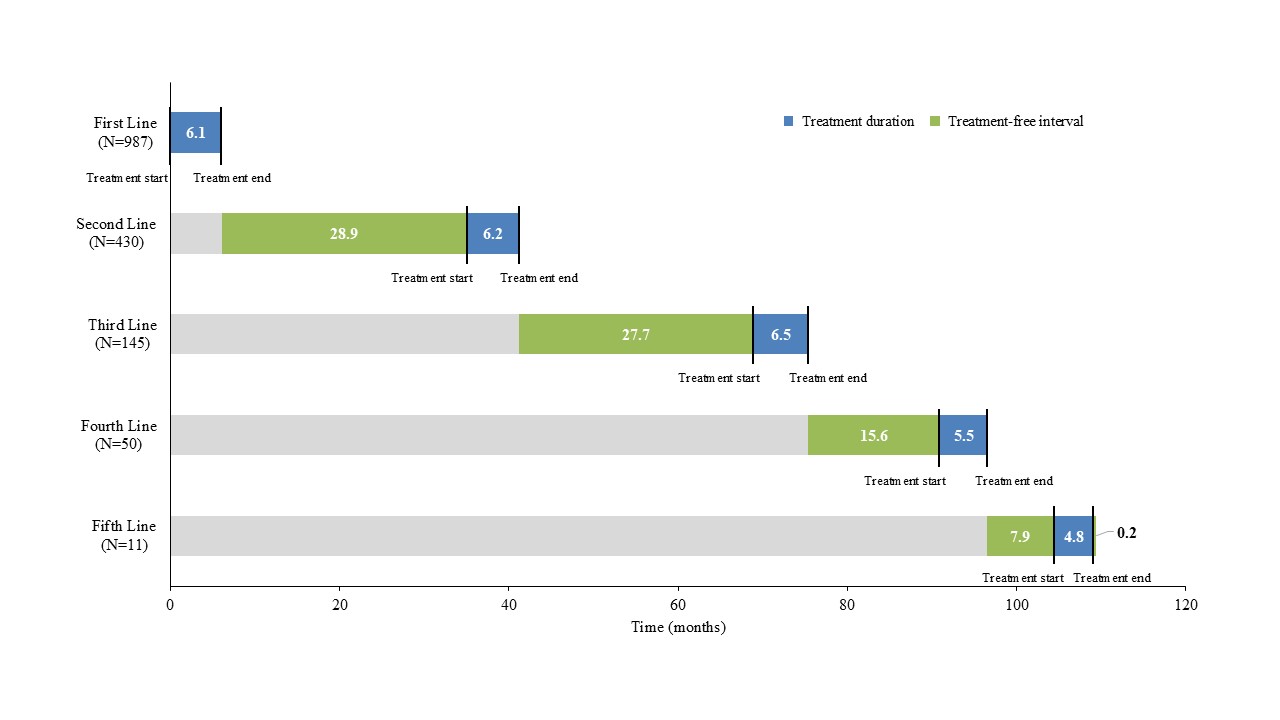

Supplement: Supplementary file 1 — Supplementary file1 (DOCX 288 KB) [file 12185_2025_3972_MOESM1_ESM.docx]
